# Efficacy of Yiqi Yangyin Jiedu decoction in postoperative patients with thyroid cancer: A protocol for systematic review and meta-analysis

**DOI:** 10.1097/MD.0000000000031803

**Published:** 2022-12-16

**Authors:** Qiao Li, Qiuju Zhang, Yili Liu, Shan Zhao, Bo Zhang, Bingxin Xie

**Affiliations:** a Department of Endocrinology, Southern Branch of Guang’anmen Hospital, China Academy of Chinese Medical Sciences, Beijing, China; b Department of Ultrasonography, Southern Branch of Guang’anmen Hospital, China Academy of Chinese Medical Sciences, Beijing, China; c Department of Surgery, Southern Branch of Guang’anmen Hospital, China Academy of Chinese Medical Sciences, Beijing, China; d Department of Spleenogastridae, Southern Branch of Guang’anmen Hospital, China Academy of Chinese Medical Sciences, Beijing, China; e Department of Cardiovascular Medicine, Southern Branch of Guang’anmen Hospital, China Academy of Chinese Medical Sciences, Beijing, China.

**Keywords:** meta-analysis, protocol, recurrence, survival, systematic review, Yiqi Yangyin Jiedu decoction

## Abstract

There is no evidence-based data to confirm the efficacy of Yiqi Yangyin Jiedu Decoction (YYJD) in postoperative thyroid cancer patients. Therefore, in order to provide new evidence-based medical evidence for clinical treatment, we used this protocol to conduct a systematic review and meta-analysis to evaluate the efficacy and safety of YYJD in postoperative patients with thyroid cancer.

**Methods:**

This systematic review and meta-analysis has been prospectively registered in the PROSPERO (No. CRD42022365826). Six databases, including Medicine, Embase, Cochrane, CNKI, Wan Fang, and VIP, will be searched from their inception to February 1, 2023. Clinical controlled studies investigating the efficacy and safety of YYJD in patients after thyroid cancer surgery will all be considered for inclusion. The primary outcomes are tumor recurrence rate and overall survival. The secondary outcomes include treatment-related adverse effects, length of hospital stay, and patient satisfaction. All data will be analyzed using R version 3.4.3 to calculate pooled standardized mean differences for outcomes. Data that can not be retrieved will be interpreted from graphs using digital ruler software.

**Results:**

The results of this paper will fill a gap in the literature regarding this project.

**Conclusion:**

We assume that the YYJD has a positive effect.

## 1. Introduction

Thyroid carcinoma is the most common malignant tumor in endocrine system. The main manifestation is anterior neck mass or thyroid nodule found in thyroid B ultrasound examination. Thyroid cancer is the fifth most common type of cancer among adolescents aged 15 to 39, and its incidence is increasing year by year.^[1,2]^ Papillary and follicular thyroid cancer, known as differentiated thyroid cancer, accounts for approximately 80 to 85% of all thyroid tumors, and its treatment depends on the stage and the presence of risk factors.^[3]^

Surgery is the standard treatment for patients with thyroid cancer. Despite the international trend towards reducing active treatment, many guidelines still recommend total thyroidectomy for patients with thyroid cancer > 1centimeter, followed by radioiodine therapy.^[4]^ This treatment strategy is associated with significant morbidity due to hypothyroidism, hypoparathyroidism, recurrent laryngeal nerve injury, halitosis and dry mouth, leading in long-term poor quality of life.^[5,6]^ The favorable prognosis of thyroid cancer indicates the overtreatment of a large number of such patients. Therefore, many scholars have tried to find alternative or adjuvant therapies, such as some herbal and botanical therapies used in cancer treatment, which may be of great importance.^[7–9]^

Chinese medicine is composed of a mixture of herbs in a single prescription, which includes multiple active ingredients that can be used for multiple targets. When combined, they can provide perfect benefits and moderate effects in a synergistic or antagonistic manner. Traditional Chinese medicine believes that the occurrence of tumors is closely related to the strength of the positive Qi in body, and that deficiency of Yin and Yang, Qi and blood, and deficiency of positive Qi are the basis of its development.^[10]^ Thyroid cancer is mostly characterized by deficiency of Qi and Yin. Qi deficiency refers to spleen-lung Qi deficiency, while Yin deficiency refers to deficiency of lung Yin.^[11]^ Based on this theory, Yiqi Yangyin Jiedu Decoction (YYJD) helps to support the positive and expel the evil at the same time. Numerous clinical studies have confirmed the efficacy of YYJD in various cancers and other chronic diseases.^[11–15]^ However, there is no evidence-based data to confirm the efficacy of YYJD in postoperative thyroid cancer patients. Therefore, in order to provide new evidence-based medical evidence for clinical treatment, we used this protocol to conduct a systematic review and meta-analysis to evaluate the efficacy and safety of YYJD in postoperative patients with thyroid cancer.

## 2. Materials and methods

### 2.1. Searching strategy

**Figure 1.**
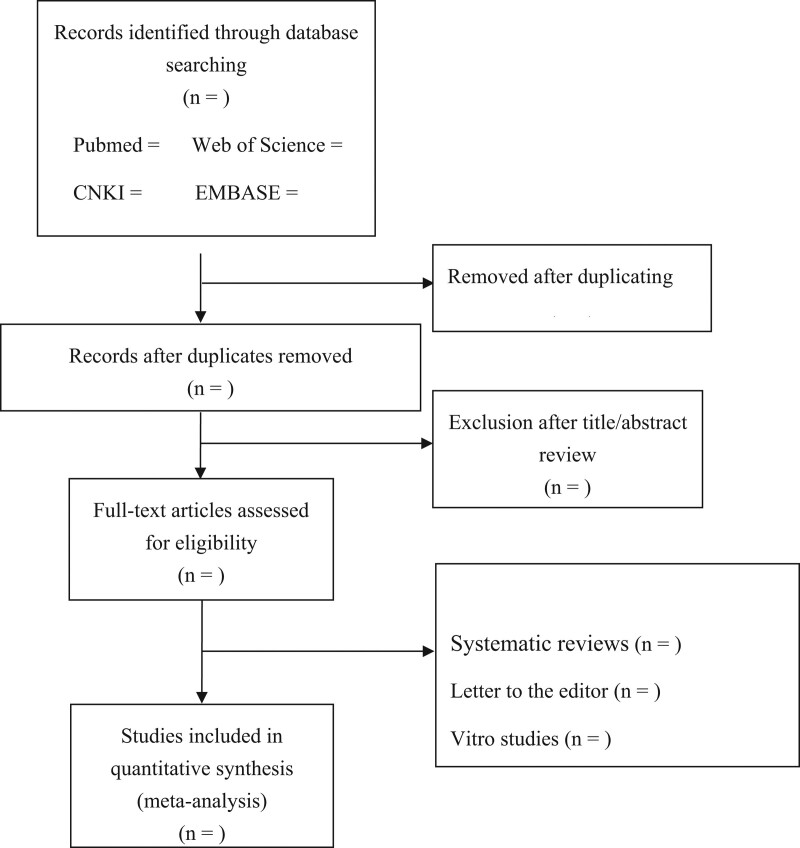
PRISMA Flow diagram describing the selection process for relevant clinical trials used in this meta-analysis.

The protocol was prepared according to the Preferred Reporting Items for Systematic Reviews and Meta-Analyses Protocols statement guidelines. This systematic review and meta-analysis has been prospectively registered in the PROSPERO (No. CRD42022365826). Six databases, including Medicine, Embase, Cochrane, CNKI, Wan Fang, and VIP, will be searched from their inception to February 1, 2023. If available, we will attempt to contact the original study authors to consult the information we need. We will perform a manual search to track down references to relevant literature. The following were the search keywords and terms we used: “thyroid cancer” OR “thyroid carcinoma” OR “thyroid adenocarcinoma” AND “Yiqi Yangyin” OR “Yiqi Huoxue” OR “Yiqi Yangyin Huoxue” OR “Yiqi Yangyin Jiedu” OR “tonifying qi and nourishing Yin” OR “supplementing qi and promoting blood” OR “nourishing Yin and promoting blood” OR “Chinese medicinal herb” OR “Traditional Chinese medicine” OR “TCM” OR “CMH” AND “clinical research” OR “cohort study.” Ethical approval is not required as the current meta-analysis will be based on previously published studies. The specific search flow chart will be supplemented as pattern in Figure 1.

### 2.2. Inclusion and exclusion criteria

#### 2.2.1. *Inclusion criteria*.

The inclusion criteria set for this paper were as follows.

Type of study: clinical randomized controlled trial or retrospective cohort study.

Type of participant: patients who underwent surgery for thyroid cancer.

Intervention group: postoperative treatment with YYJD on top of conventional treatment.

Control group: postoperative treatment with conventional therapy only.

Outcome measurements: primary outcomes are tumor recurrence rate and overall survival; secondary outcomes include treatment-related adverse effects, length of hospital stay, and patient satisfaction.

#### 2.2.2. *Excluding criteria*.

The following will be excluded: non-cohort trials; detailed data on outcomes were not reported or could not be calculated from the data provided; studies were poorly designed for comparisons; serial publications from the same cohort, with overlapping participants and study designs, also excluded.

### 2.3. *Study selection and data extraction*

After removing duplicate search terms, 2 reviewers will screen titles and abstracts for studies that meet the eligibility criteria above. Subsequently, the remaining records will be submitted for full-text evaluation. During this step, the reference list of each manuscript will be reviewed for relevant citations. All electronic database screening, data extraction, and quality assessment will be performed in duplicate by 2 independent researchers. Differences that cannot be resolved after mutual discussions and revisions will be considered by the third researcher.

A customized data extraction table will be developed for the outcome. The lead author useds only published data for data extraction. The following information is mandatory: authors, year of publication, number of patients assigned to the YYJD treatment and control groups, patient characteristics such as age, gender, BMI and ethnicity, treatment details such as duration, treatment status and drug dose, follow-up period and outcome measures. For meta-analyses of data, means and standard deviations will be obtained from original papers or contact authors. Data that can not be retrieved will be interpreted from graphs using digital ruler software. For quality assessment purposes, studies will be classified by study design.

### 2.4. Statistical analysis and data synthesis

All data will be analyzed using R version 3.4.3 to calculate pooled standardized mean differences for outcomes. If the study reported the effectiveness of the aromatic essential oil at multiple time points, the last time point is used in the analysis. Because of the different conditions in the same outcome, the corresponding 95% confidence intervals for the pooled effect size are calculated using a random effects model. Heterogeneity is assessed with the *Q* statistic and is considered significant when *I*^2 ^> 50% or *P* < .1. Publication bias is assessed using Egger’s regression test and graphically represented using Begg’s funnel plot when there are ≥ 10 studies. In Egger’s regression test, *P* < .10 is considered significant. Whenever publication bias is discovered, Duvall and Tweedie’s pruning and padding approach is applied to supplementary studies that seemed to be missing to enhance symmetry.

### 2.5. Risk of bias

The Cochrane risk of bias tool will be independently used to evaluate the risk of bias of included randomized cohort studies by 2 reviewers. The quality will be assessed by using following 7 items: random sequence generation, allocation concealment, blinding of participants and personnel, blinding of outcome assessment, incomplete outcome data, selective reporting, and other bias. A modified version of the Downs and Black tool is adopted to evaluate the quality of non-randomized cohort studies. The modified version consists of 27 items with a total possible score of 29. A score of ≥ 75% indicates high quality, 60% to 74% indicates moderate quality and ≤ 60% low quality. Two investigators independently evaluate included studies on the 27 criteria, with any discrepancies resolved by a third independent reviewer.

## 3. Discussion

With the continuous promotion of the treatment concept of integrated traditional Chinese and Western medicine, the comprehensive treatment scheme of surgery combined with traditional Chinese medicine is gradually adopted in the conventional anti-cancer process. We hypothesize that YYJD can improve the symptoms of Qi and Yin deficiency in patients after thyroid cancer resection to a certain extent and improve the immune status of patients, thus improving their survival quality. Future randomized controlled trials with higher quality and larger sample sizes are needed to further reveal its efficacy. Meanwhile, research on the related mechanism of action needs to be strengthened to provide a basis for promoting the formula in clinical application. There are also foreseeable limitations of this study: Since the formula was first developed in China, most of the included literature may originate from China, which may pose some risk of bias; The sample size of the included studies was limited; There may be mild differences in the herbal protocols used in each study.

## Author contributions

**Conceptualization:** Yili Liu, Shan Zhao.

**Data curation:** Qiao Li, Qiuju Zhang.

**Investigation:** Qiao Li, Qiuju Zhang.

**Methodology:** Yili Liu, Shan Zhao.

**Funds:** Bingxin Xie.

**Software:** Shan Zhao.

**Supervision:** Yili Liu.

**Writing–original draft:** Qiao Li, Qiuju Zhang.

**Writing–review & editing:** Bo Zhang, Yili Liu.
